# A qualitative study of Western Australian women's perceptions of using a Snoezelen room for breastfeeding during their postpartum hospital stay

**DOI:** 10.1186/1746-4358-3-20

**Published:** 2008-08-13

**Authors:** Yvonne L Hauck, Lisa Summers, Ellie White, Cheryl Jones

**Affiliations:** 1 School of Nursing and Midwifery, Curtin University of Technology, Perth, WA, Australia; 2 Women and Newborn Services, Osborne Park Hospital, Osborne Place, Stirling, WA, Australia

## Abstract

**Background:**

There is limited evidence on the use of the Snoezelen concept for maternity clients. Snoezelen, a Dutch concept, initiated in the 1970s as a leisure activity for severely disabled people, involves creating an indoor environment using controllable stimuli to enhance comfort and relaxation. These specially designed rooms expose the user to multiple sensory stimulations combining vision, touch, sounds and aromas. The aim of this study was to provide insight into breastfeeding women's experience of using a Snoezelen room during hospitalisation.

**Methods:**

A qualitative exploratory design was chosen to reveal women's perceptions of using the Snoezelen room. Osborne Park Hospital, the study setting is the second largest public provider of obstetric services in Western Australia. A purposive sample was drawn from breastfeeding women who used the Snoezelen room during their postpartum stay from March 2006 to March 2007. Saturation was achieved after eleven breastfeeding women were interviewed six weeks post discharge. Data analysis involved the constant comparison method.

**Results:**

Participants entered the room feeling tired and emotional with an unsettled baby and breastfeeding issues aggravated by maternal stress and anxiety. All women indicated they were able to achieve relaxation while in the room and would recommend its use to other breastfeeding mothers. Two key themes revealed how the Snoezelen room facilitated maternal relaxation, which ultimately enhanced the breastfeeding experience. The first theme, "Finding Relaxation for the Breastfeeding Mother" incorporates three subthemes: 'Time out' for mother; Control in own personal space; and a Quiet/calm environment with homelike atmosphere. The second theme, "Enabling Focus on Breastfeeding", occurred after relaxation was achieved and involved four subthemes: Able to get one-on-one attention; Not physically exposed to others; Away from prying, judgemental eyes and Able to safely attempt breastfeeding alone knowing help is nearby.

**Conclusion:**

Insight into how the Snoezelen room promoted relaxation also highlights what contributes to maternal anxiety during breastfeeding experiences in hospital. The findings offer health professionals the opportunity to consider adopting strategies such as a Snoezelen room in their hospital or being innovative in modifying the postpartum setting to promote relaxation for breastfeeding women.

## Background

Breast milk is recognised as the ideal nutrition for the human infant. Research continues to explore supportive strategies to enhance women's initiation and prevalence of breastfeeding. Although Western Australia had initiation rates as high as 84% in the mid 1990s [1], evidence suggests that initiation rates have continued to increase to 88% [2] and more recently to 94% [3]. Prevalence rates, however, are not as encouraging as rates decrease to 62% at 3 months and 50% at 6 months [2]. Western Australian data is comparable to national figures from the 2001 National Health Survey with 87% of infants receiving some breast milk, 48% being breastfed to six months, and 23% to 12 months [4]. Early cessation has been attributed to ineffective support from health care professionals and informal networks such as family and friends, unrealistic expectations, physical concerns with breastfeeding and faltering commitment by mothers [5]. The most common reason cited for stopping breastfeeding within the first two weeks post birth was an unsettled baby, which mothers interpreted as indicating insufficient milk supply [6].

Adequate support for the breastfeeding woman is essential to promote initiation [7]. Support during breastfeeding initiation focuses upon ensuring that the woman can achieve correct attachment, understands the principles of supply and demand, and receives prompt treatment for any problems. Therefore, the support of a skilled, competent midwife during the initiation phase of breastfeeding cannot be underestimated.

Snoezelen is a concept whereby an indoor environment using controllable stimuli is created to provide comfort. The specially designed room exposes the user to multiple sensory stimulations combining vision, touch, sounds and aromas. These rooms are credited with providing positive therapeutic or educational effects and positive emotions such as well-being, rest, satisfaction, poise and joy [8]. The original purpose of the Snoezelen concept was as a leisure activity for severely disabled people. Its popularity has grown since the 1980s beyond the original counties of Germany and the Netherlands, to Britain, Canada, the United States, Spain and Australia with use extending beyond mentally impaired clients. This environment has been used with adults with cerebral palsy [9]; multiple handicap clients [10]; nursing home and psychogeriatric clients [11-16]; palliative day-care clients [17]; end-stage Alzheimer's clients [18]; neonates [19]; critically ill children [20]; and chronic pain sufferers [21-23].

There is limited evidence on the use of the Snoezelen concept with maternity clients. One study has reported how using the Snoezelen room enhanced the labour experience of Western Australian women by providing distraction, environmental control, comfort, relaxation, choice of complementary therapy features and safety in a non-clinical atmosphere [24]. Consequently, this research adds to a new body of knowledge by providing insight into the experience of using a Snoelezen room with maternity clients and specifically, breastfeeding women.

## Methods

### Research aim

The aim of this study was to provide insight into the experience of using a Snoezelen room for breastfeeding women in the early postpartum period.

### Research design

A qualitative exploratory design was employed to obtain a rich description of the experience of using the Snoezelen room. Therefore, a small cohort of breastfeeding women was invited to participate in in-depth interviews after using this environment. The average length of hospital stay for maternity clients is 2.7 days and to conduct an interview during this limited hospital stay when clients are in the acute phase of recuperation was not feasible. Therefore a time period of 4 to 6 weeks was chosen to balance the need for initial recuperation from the birth process and potential concern with recall bias.

### Setting and context

**Figure 1:**
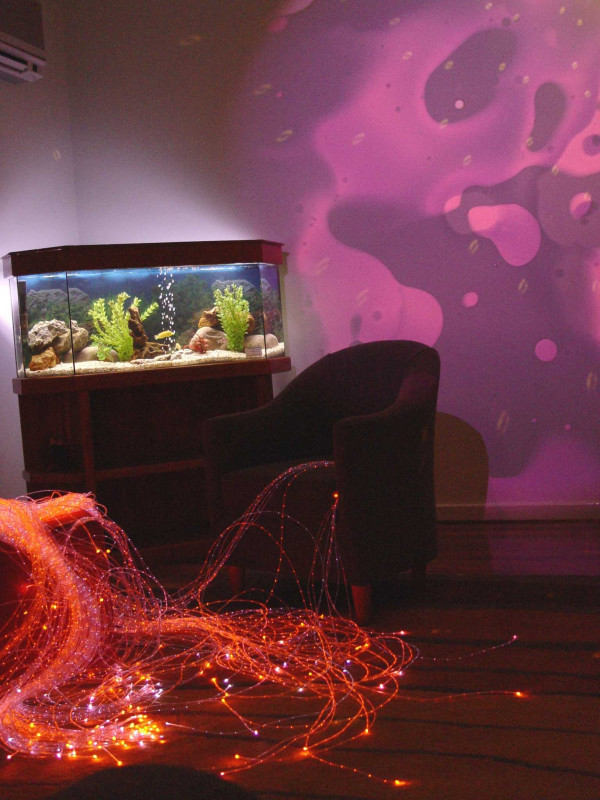
Snoezelen room at Osborne Park Hospital.

Osborne Park Hospital (OPH), the setting for this study is the second largest public provider of obstetric services in Western Australia. The maternity setting at OPH is considered a low to moderate risk maternity unit with approximately 1600 births per year. Healthy women qualify to birth at OPH after they have reached 35 completed weeks gestation. The Snoezelen room at Osborne Park Hospital was designed especially for the maternity setting (Figure 1). The midwives, who introduced the concept into the maternity setting, took initial guidance from the website of the International Snoezelen Association [8] where the concept definition and aim is provided. However, Snoezelen rooms are unique as they are based upon a target audience. The room has polished floorboards and soft earthy colours of green, brown, terracotta and yellow. These colours were chosen as the midwives felt they represent the link between birthing and nature. The room provides a three-seater lounge with a chaise where a woman can lie and relax with a chair with a wrap-around backrest. There is a large soft rug and three-square ottomans for women to put their feet up while sitting on the lounge or chair. The main features of the room are the wheel projection that slowly rotates to display patterns on the wall and fibre optic lights that can be draped across a person or the room as it gradually changes colour. Finally, a tropical fish tank, music and aromatherapy were chosen to complete the room's ambience.

### Recruitment

The sample for this study was drawn from breastfeeding women at Osborne Park Hospital (OPH) who chose to use the Snoezelen room for breastfeeding reasons during their hospital stay. Information about the Snoezelen room and its aim to promote relaxation is provided during hospital tours, antenatal classes, and via posters displayed in every birth room, the patient lounge and all postpartum rooms. All client requests to use the room are respected depending upon the room's availability. Midwives tend to encourage use of the room for relaxation for breastfeeding women, women in early labour, tired and anxious women needing a sleep and pregnant women with elevated blood pressure who are awaiting a blood pressure profile assessment. Only one client is able to use the room at a time and a midwife must unlock the door to provide access.

All midwives on the postpartum ward at Osborne Park Hospital who put a woman in the Snoezelen room for breastfeeding issues were asked to enter the woman's name and date of the visit in a book. Three weeks after using the Snoezelen room an information letter was posted to 44 women who used the room for breastfeeding between March 2006 and March 2007. Potential participants replied to the letter by returning an expression of interest form to the researcher in a reply paid envelope. Twelve women replied to the invitation and eleven were interviewed. One woman had not used the room for breastfeeding but to gain some privacy and much needed sleep and was therefore not interviewed. The researcher contacted interested women to arrange an interview. This was to avoid perceptions of coercion should hospital midwives approach the women. Arrangements were made to conduct the interview in the privacy of the woman's home. The researcher who is a midwife but not involved in client care at the hospital conducted the interviews. This purposeful sampling method ensured that participants selected were appropriate and able to share perceptions of using the Snoezelen environment for breastfeeding issues [25]. Participants received an information letter and provided informed consent while being assured they could withdraw from the study at any time. An interview guide with five open-ended questions was used to encourage the women to share their experience: "tell me about your breastfeeding while in hospital"; "tell me about using the Snoezelen room for breastfeeding"; "what did you like/dislike about the Snoezelen room" and "would you recommend the room to other mothers". The interviews lasted between 45 minutes and 90 minutes (average of 60 minutes) as the questions facilitated a "story telling" atmosphere. Prompt questions were also used such as "can you elaborate on that" or "tell me more about". All interviews were audio-taped and transcribed verbatim. A demographic form was also completed to provide a brief profile of the participants (i.e. age, type of birth, education level, current breastfeeding pattern, number of children breastfed, breastfeeding intention). The final sample size of 11 participants was determined by data saturation as ongoing analysis revealed redundancy of information and no further concepts or themes were apparent [25].

### Data analysis

The constant comparison method modified from the grounded theory methodology was used to analyse the interview transcripts [25]. Categories drawn from the data were compared to reveal commonalities and variations in the experience [26]. Data analysis identified patterns or themes relevant to the mothers' experience of using the Snoezelen room. Initially team members conducted a separate data analysis with the transcripts and then came together for discussions to clarify, negotiate and refine the findings. Disagreements on interpretation were negotiated and although major disagreement did not occur, transcripts were referred back to for refinement of final themes. An audit trail was kept by the first author to provide transparency of the decisions and allow evaluation of how data were categorised into themes and subthemes [26]. To ensure trustworthiness by confirming validity of the findings, a summary of the identified themes was posted to participants inviting comment and discussion [26]. Due to changes in postal addresses and phone numbers over the course of the study period, four participants could not be contacted. The remaining seven participants confirmed that the themes were an accurate reflection of what the Snoezelen room offered a breastfeeding woman.

### Ethical considerations

Ethical approval was obtained from the hospital and university human ethics and review committees. Data were coded with numbers to ensure confidentiality. Transcripts and demographic data forms were stored in a locked cabinet at the university. The person employed to transcribe the cassettes signed a confidentiality agreement to ensure they would not breach confidentiality and were aware of the seriousness of this issue. If participants become distressed during the interview process, a referral system was prearranged with appropriate counselling services. Referral was not undertaken as no women experienced any distress during the interviews.

## Results

It must be acknowledged that the sample was self-selecting and not representative as not all breastfeeding women chose to use the room. In addition, only 11 out of the 44 women invited agreed to participate in the study. All participants interviewed felt they benefited from using the Snoezelen room; however, we have no information regarding the women who chose not to participate. They may not have found the room helpful or had ceased breastfeeding when the invitations were posted, although the information letter stressed that their current infant feeding pattern was not relevant to the study. Finally, conducting an interview six weeks post birth could have resulted in recall bias, however, periods of less than three years for recall with infant feeding are considered acceptable [27].

Eleven women shared their experience of using the Snoezelen room during the early stages of their breastfeeding. All women had a positive experience in the Snoezelen room and indicated they would not only use it again but would recommend it to other mothers. Seven women were first time breastfeeding mothers, with three women breastfeeding their second child and one breastfeeding her third. The age of the mothers ranged from 23 to 39 years with a mean of 31 years. The length of hospital stay during the postpartum period ranged from 1 to 8 days with a mean of 4 days. All participants were living with a partner. The time of the interview ranged from 4 to 11 weeks post birth with a mean of 6.5 weeks. Ten of the eleven participants were still breastfeeding their infant at the time of interview. The mother who was completely formula feeding at her 11-week interview had planned to breastfeed for between 4 to 6 months. Seven mothers used the Snoezelen room once during their hospitalisation, two used it twice and two used the room for three or more times. The time spent in the room ranged from 30 minutes to 8 hours with the majority of women using the room for 1 to 2 hours for each visit. Four mothers specifically asked to use the room and three of these women had used the Snoezelen room during their labour. The remaining women used the room on their midwife's recommendation. The majority (n = 9) used the room for an unsettled baby and/or breastfeeding issues. One woman, who used the room during labour and did not have an unsettled baby but enjoyed the room so much she used it for several visits during her hospital stay and for up to 8 hours for one visit. Finally, one woman was not having breastfeeding difficulties but was unwell after experiencing a postpartum haemorrhage. Her midwife encouraged use of the room for relaxation and the woman did use this opportunity for breastfeeding. Further demographic data are provided in Table 1.

**Table 1:** Demographic profile

**Demographic variables**	**Participants** ** (n = 11)**
**Type of birth**	

Spontaneous vaginal birth	6
Forceps or vacuum birth	1
Caesarean birth	4

**Educational level**	

University	7
Technical and further education	1
High school certificate	2
Completed year 10 in high school	1

**Feeding pattern during interview**	

Completely breastfeeding	8
Breastfeeding with occasional/regular bottle of expressed breast milk	1
Breastfeeding with occasional/regular bottle of formula	1
Completely formula feeding	1

**Plans for breastfeeding**	

< 1 month	
1 to 3	
4 to 6	3
7 to 9	1
10 to 12	3
> 12	3
Uncertain	1

The majority of women who chose to use the Snoezelen room by asking or following their midwife's recommendation were experiencing anxiety due to breastfeeding issues. Most were day two or three after their birth and were tired, emotional and having breastfeeding difficulties with attachment or pain with feeding. The majority of mothers had infants they described as unsettled. The three women who had used the Snoezelen room during labour asked to use the room earlier than the other participants. Although all postpartum rooms have a poster of the Snoezelen room only one mother who had not used the room in labour asked to use the room. The majority of women indicated that they would have never thought to ask about the room even though they were aware of it.

All participants indicated that they were able to achieve some relaxation while in the room. Two key themes with seven subthemes were revealed during data analysis highlighting how the Snoezelen room enhanced relaxation for these breastfeeding mothers (Table 2).

**Table 2:** Key themes and subthemes

**THEME: Finding Relaxation for the Breastfeeding Mother**
"Time out" for the mother
Control in own personal space
Quiet/calm environment with homelike atmosphere

**THEME: Enabling Focus on Breastfeeding**

Able to get one-on-one attention
Not physically exposed to others
Away from prying, judgemental eyes
Able to safely attempt breastfeeding alone knowing help is nearby

Participant quotes will be provided to illustrate each of these themes. Participants are identified by a code P1 to P11 to demonstrate the depth and variety of experiences and illustrate how all women are represented. The first theme was "Finding Relaxation for the Breastfeeding Mother" which incorporated three subthemes: Time out for mother, Control in own personal space and Quiet/calm environment with a homelike atmosphere.

### Time out for mother

Being in the Snoezelen room was an escape from the ward environment and mothers appreciated being able to get away from the noise and activity. The room provided the opportunity to have a break from other mothers and their crying babies, visitors, and the hospital staff, no matter if they were doctors and midwives or the domestic staff doing their cleaning duties. Having private time meant having the freedom to do what she wanted such as put her feet up, listen to music or even have a cry in private. As one mother stated, "*I did sit there for a little while and have a good cry. I thought that I was doing it [breastfeeding] wrong, I thought it was a problem with me. I couldn't do it, I did sit there and have a little time out (P7)."* Having time out promoted relaxation for the women. Although all of the participants had their babies with them and were therefore not alone, they perceived that being in the room provided a haven or escape from being in the traditional maternity setting. One woman described how the room was not only a break for her but how her daughter enjoyed the lights in addition to breastfeeding well: "*She was just eyes wide open watching all the colours change; just really still and quiet and loved it; I fed her in there and I found it much nicer and just a break for me as well (P3)."*

Three women had a midwife come in the room for some of the time to assist with breastfeeding. Four women had their partners in the room, but only one mother encouraged visitors to come into the room. Having time out for the mother illustrated how being in the room was seen to be an indulgence: *"Nice to just have that little bit of peace, no I don't think I would have tried with anybody else in there, maybe my mum but I don't think I would have taken the kids or anyone else, selfish, I save that for me (P8)."*

### Control in your own personal space

The second theme highlighting how relaxation was enhanced focused upon having control of personal space and who entered that space. Even being in a private room did not afford women control. Health care professionals and hospital domestic staff continuously entered women's rooms. Closed doors and drawn curtains were no guarantee of privacy. *"There were a few occasions where I was feeding and obviously the people would go 'Oh no don't mind me', I'm thinking you might not mind but actually this is quite a personal thing for me and I'm not really comfortable with it (P6)."* The Snoezelen room door requires a key to enter or the visitor to knock to be allowed in. Most women indicated that it was rare for anyone to disturb them while in the room and the sign on the door indicating the Snoezelen room was in use also contributed to respect for privacy. *"The room was a safe haven, the privacy to do what I needed to do and to not have to worry about conforming to what they wanted me to be like in the ward (P7)."*

The modifiable features in the room such as movable furniture, music and lighting options were also a desirable feature that promoted control and therefore, relaxation. Traditional hospital rooms do not allow the flexibility of dimming lights or chairs with comfortable arms and footstools that can be moved to personal preferences. *"I was stunned because I think I just felt that as soon as I got in there and sat down and knew that I wasn't going to be bothered by anything, I wouldn't hear anybody else around me, I could set the environment how I wanted it, I just automatically felt myself relax and just chill out (P10)."*

### Quiet/calm environment with homelike atmosphere

The quiet and calming atmosphere in the Snoezelen room created feelings of safety plus the comfortable furniture was seen to provide a more homelike ambience. *"It was just so nice to have our own little room where you're warm and cozy and feel safe, I did feel safe in there (P7)."* The mothers commented on how their emotional and physical state of relaxation transferred to their unsettled babies whose behaviour improved. *"He [baby] seemed a lot more settled in there, I assume purely cause I was and I could get comfortable, I found the beds very uncomfortable for feeding and the chairs are ok, but it [Snoezelen room] was just really nice, you feel like you're at home (P5)."* The mean hospital stay for these women was 4 days, which is longer than the setting average of 2.7 days. Having the opportunity to go into a quiet and calm environment was welcomed by these women who accepted their need to stay in hospital for individual reasons but appreciated having a sanctuary from the ward. *"The privacy as well was a huge bonus to know that you didn't have the kids peeking in through the curtains or the person delivering your meal or the nurse coming in to check your bits and pieces, so it was good to be in that home environment (P11)."*

The second key theme "Enabling Focus on Breastfeeding" occurred after the mother was able to achieve some relaxation and encompasses four subthemes: Able to get one-on-one attention; Not physically exposed to others; Away from prying, judgemental eyes; and Able to safely attempt breastfeeding alone knowing help is nearby.

### Able to get one-on-one focused attention

Those mothers who did require the support of a midwife in the Snoezelen room with breastfeeding commented how being in the room afforded them one-on-one focused attention. They felt that in a shared maternity room, they were not able to achieve this level of attention, due to the presence of other mothers and babies, partners, families and visitors. When a midwife joined the mother in the Snoezelen room there were no bells or buzzers around to distract the midwife or take her away and even if the time wasn't long, the undivided attention was valued. As one mother shared: "*It was really nice to go into a separate space and sit down with someone to really focus on trying to get the breastfeeding right... really good to have that special attention (P2)."* Being more relaxed in the room due to the privacy and atmosphere also contributed to the mother being better able to calmly listen and take in the advice being offered. *"If you needed one of the midwives to get them to come in there which was nice, cause you just sit and relax on the sofa as you would at home instead of sitting on the end of a bed or on a chair or something, so it was a lot easier and a lot calmer (P4)."*

### Not being physically exposed to others

Not all women feel comfortable having their breasts exposed as may occur when trying to initiate breastfeeding in a hospital setting. The women using the Snoezelen room indicated how they appreciated the privacy and not having to worry about *"covering up" *while attempting to breastfeed. The door to the Snoezelen room was locked and midwives tended to knock before they entered the room, even though they had a key. This respect for privacy was noted as being different from the traditional hospital room, even if the woman was in a private room. *"When the person next door came actually into our section, it was like 'Oh my God, I'm sitting here with my breast hanging out', which wasn't very nice (P4)."* The mothers commented that they felt confident that no one was going to come in unannounced while they were trying to breastfeed. This privacy was like being in your own home and was appreciated. *"The privacy of the breastfeeding which is a very intimate experience that I'm not that sort of outgoing that I would enjoy exposing myself publicly (P6)."*

### Away from prying judgemental eyes

Many women commented upon their feelings of being judged by others regarding either difficulties with breastfeeding or just the fact that their baby was crying, unsettled and disturbing others. *"I did find that the room took away my tension and the stress of not being able to do it, it did relax me, there was no one to watch me and see how bad a job I'm doing (P7)."* The sensitivity to being seen and judged was not just with regards to other mothers, partners and visitors, it also included the evaluating and watchful gaze of health professionals: *"You don't want to be judged and it sounds a bit strange as helpful as the midwives were when you are trying even when I was feeding they'd come in and go 'Is she attached properly?' and then come and have a look and check (P6)."* Feeling like they had to perform in front of others placed added stress to an already anxious woman whose breastfeeding expectations were not being realised. Being away or not being under the scrutiny of others, did help to ameliorate some of this anxiety: *"Completely away from everyone else, because I found it very stressful trying to do things even though everybody's baby was crying and everybody's having issues you still think you're the only one and you think you're the problem and it's just nice to be able to go away and not have anyone listening to you (P9)."* The Snoezelen room provided a safe retreat: *"Somewhere where eyes aren't going to be looking at you and asking 'How are you going, can you do it right or can you not', it's just your little space (P1)."*

### Able to safely attempt breastfeeding alone knowing help is nearby

Having an unsettled baby as a consequence of breastfeeding difficulties, meant that many of these women commented how their confidence was slowly eroding away. *"I'd gotten to a point where I was saying to my husband 'No that was it. I am not doing this. It's not worth it'. Thank goodness I didn't but I was losing confidence (P10)."* To regain confidence it was important to have a safe environment to try breastfeeding strategies for attachment as one example and know that help was available. Ultimately, the mothers wanted to be able to achieve a level of competency with their breastfeeding that could be transferred to their hospital room and home once discharged. The Snoezelen room provided this opportunity: *"I wanted to experiment and explore it on my own because I'm not a complete idiot, sometimes you just need a chance to try it for yourself. You want some time out to yourself to see if you can do it yourself and I knew that in that space I was just going to have some time to find out for myself and not have any interruptions (P6)."*

### Consequences

Using the Snoezelen room was described as a turning point for a number of participants during their early breastfeeding experience. The opportunity to use the room while initiating breastfeeding was described as a positive experience. Being able to achieve a degree of relaxation assisted most of the women in being able to have a positive breastfeeding experience. For some women, this one affirming experience contributed to a journey toward breastfeeding success. *"I just think knowing that I could do it and knowing what it felt like to have that right positioning and that feeling so that if she wasn't on properly I knew it (P11)."* A number of mothers said the positive experience increased their confidence in their ability to breastfeed and reinforced the idea that: *"I can do this."* Prior to coming into the room, many women were emotionally and physically distressed. Their baby was unsettled and breastfeeding problems compounded the situation. *"I was losing confidence. I was definitely not doing it right. It was a turning point for me. I quite enjoy breastfeeding now. I am glad I didn't give it up (P10)."*

As the mothers relaxed, so did their babies and the majority of women were able to breastfeed successfully in the room. One woman's baby was not interested in feeding in the room even though she tried but her previously unsettled baby did settle and was content in the room, which reinforced to the woman how important it was to relax. Still another woman who breastfed with continuing nipple pain was satisfied that the experience was worthwhile and has continued to receive specialist support from a community lactation consultant at six weeks postpartum. Having a relaxed baby was a key issue for these women who noted a difference in their baby's behaviour. *"Every time we were in there he was really calm, slept really well and breastfed really well and when we went down to the [hospital] room a few times he'd start howling so we went back down to the [Snoezelen] room and he'd calm down, we think that everybody should have a Snoezelen room at home (P4)."* All babies settled while in the room and some mothers and babies even managed to have a nap in the Snoezelen room before returning to their hospital room. Women commented how having a positive breastfeeding experience left them feeling refreshed and rejuvenated and most importantly better able to cope with the challenges that lay ahead. One woman's comment aptly highlights her summary of the Snoezelen room: *"It was just such a blissful place, just missing chocolate really (P9)."*

## Discussion

Our findings highlight what assisted breastfeeding mothers to achieve relaxation within a Snoezelen environment, which ultimately facilitated their early breastfeeding experience. It is anticipated that the rich description of these qualitative findings will enable the reader to determine the transferability of the findings to their own context [25,26]. Although limited maternity settings may actually have a Snoezelen room, application of how the room enhanced relaxation can be considered and addressed in other settings. A woman who is anxious, in considerable pain or distressed due to physical concerns may find it difficult to relax during breastfeeding. The milk ejection reflex is enhanced when the woman is comfortable, relaxed, and not experiencing undue pain or anxiety [28]. Measures to enhance relaxation such as adequate pain relief and the provision of a calming environment may be a complementary strategy to midwifery support for breastfeeding women. The provision of a calming environment that addresses issues such as ensuring privacy and a space for the mother to have "time out" and control, feeling safe from prying, judgemental eyes, and having the opportunity of one-on-one focused support could be creatively considered in different maternity settings.

Findings revealed factors that were perceived as stressors for new mothers such as limited access to privacy, wanting to do their best by breastfeeding and being a 'good mother' but feeling vulnerable to being judged. The link between breastfeeding and being seen as a 'good mother' has been noted [29,30]. "Heading toward the new normal" has been described as the process women undergo in the early postpartum period while reorganising life as a mother [31]. Becoming competent and developing confidence are two components of the settling in to this "new normal". Dykes' [32] study also confirmed that gaining confidence in the skill of breastfeeding was regarded as a mother's primary goal and how having a discontented newborn resulted in the mother becoming anxious and doubtful of her abilities. Ideally, a focus of postpartum care should be to foster the confidence that new mothers are struggling to achieve in those early days of breastfeeding and not present obstacles that undermine this developing confidence.

Participants indicated how their breastfeeding challenges were threatening their confidence but how having a positive breastfeeding experience assisted in boosting their faltering confidence. Uncertainty and threats to maternal confidence have been regarded as key concepts in women's breastfeeding experience [33]. However, building confidence and reducing uncertainty in being a 'good mother' by successfully breastfeeding is not a simple process. Uncertainty and vulnerability have been noted as key issues for women who encounter initial breastfeeding difficulties and reality isn't meeting expectations [33,34]. However, most women are not prepared to experience difficulties with breastfeeding, when in fact the evidence suggests that 83% of Perth women experience one or more problems during the early stage of their breastfeeding [6]. Therefore, encouraging women to have realistic expectations regarding initial difficulties while ensuring appropriate support is available to overcome these difficulties is recommended for health professionals advocating breastfeeding.

Most participants in this study entered the Snoezelen room with an unsettled baby. Given the most common reason for stopping breastfeeding within two weeks post birth was an unsettled baby, interpreted as an inadequate milk supply, anxiety over supply is a serious issue as it is associated with early cessation of breastfeeding [6]. Postpartum anxiety has been associated with reducing breastfeeding confidence [35]. Physical challenges have also been noted as affecting a woman's relationship with her newborn with some women being reluctant to continue breastfeeding due to the feelings of physical vulnerability, pain and discomfort [36].

Breastfeeding self-efficacy involves the mother's perception of her ability to breastfeed with higher self-efficacy being associated with longer duration [37,38]. The tendency to experience negative emotions, such as anxiety, depression and irritability, can impact cortisol regulation and vulnerability to stress [39]. In fact, breastfed infants exposed to higher cortisol levels in breast milk demonstrated temperament changes such as increased fear behaviours [40]. Therefore, strategies that focus upon addressing maternal anxiety, enhancing confidence and promoting breastfeeding self-efficacy have potential benefits to both mother and infant.

## Conclusion

Health professionals make a difference to breastfeeding. Their encouragement and support is associated with longer duration and greater exclusive breastfeeding rates [38]. Awareness of how early breastfeeding issues can influence maternal anxiety and breastfeeding confidence allows the health professional to better support vulnerable women experiencing anxiety due to early breastfeeding issues. Fostering maternal relaxation in an environment like a Snoezelen room is just one example that may be considered to accompany the support already provided by midwives working in postpartum settings. A comfortable, relaxed mother with adequate midwifery support is more likely to successfully initiate breastfeeding, have a settled infant and develop the confidence she needs to continue breastfeeding after leaving hospital.

## Competing interests

The authors declare that they have no competing interests.

## Authors' contributions

YH designed the study, recruited and interviewed participants, analysed data and drafted the manuscript. LS, EW and CJ assisted in analysis of the data and provided constructive feedback during revisions of the manuscript.
